# Morphological Design and Synthesis of Nanoparticles

**DOI:** 10.3390/nano14040360

**Published:** 2024-02-15

**Authors:** Mirela Honciuc, Andrei Honciuc

**Affiliations:** “Petru Poni” Institute of Macromolecular Chemistry, Gr. Ghica Voda Alley 41A, 700487 Iasi, Romania

Nanoparticles are particles with dimensions measured in nanometers, and exist at a scale where the physical, chemical, and biological properties of materials can differ significantly from those at a larger scale. Their unique characteristics are not merely due to their small size, but also arise from their high surface area to volume ratio, quantum effects, and the specific arrangements of their atoms.

The importance of nanoparticles in science and technology cannot be overstated, with numerous advancements being made across a wide array of disciplines, including medicine, electronics, energy, and environmental science. In medicine, nanoparticles are used for targeted drug delivery systems, which allow for the direct treatment of diseased cells while minimizing damage to healthy ones, significantly improving therapeutic outcomes. In electronics, they enable the development of smaller, more efficient, and more powerful devices. In the realm of energy, nanoparticles contribute to the creation of more efficient solar cells and batteries. Moreover, in environmental applications, they are used for pollution remediation and water purification, showcasing their versatility and utility in addressing some of the most pressing global challenges. In this Special Issue, we have carefully selected contributions that address all of these key aspects of the advanced applications of nanoparticles.

*Nanoparticles are at the forefront of biomedical innovations and drug delivery systems*. This is demonstrated by Sysak et al. [1] in their review article, which explores the subject of the synthesis and characterization of metal nanoparticle–flavonoid conjugates, emphasizing their potential to enhance bioavailability and target specificity in medical applications. The combination addresses the limitations of flavonoids, such as poor solubility and rapid metabolism, by leveraging the unique physicochemical properties of metal nanoparticles. The discussion spans various synthetic strategies, physicochemical properties like size and surface charge, and the biological implications of these hybrid materials, particularly their applications in cancer therapy, immune modulation, and as potent antioxidants. Yet another contribution from the forefront of nanoparticle applications is the review by Chaves et al. [2] that highlights the potential use of liposomes, which are soft nanoparticles/biomimetic particles, as versatile drug delivery vehicles for encapsulating bioactive compounds for improved stability and efficacy. Liposomes are intricate vesicular structures formed by one or more phospholipid bilayers separating the exterior aqueous medium from the interior one, with diameters ranging from 20 nm to several microns. This systematic review highlights the burgeoning application of liposomes in both cosmetics and food industries, focusing on the encapsulation of vitamins for enhancing their bio-accessibility and bioavailability, thus bridging the gap between pharmaceutical and food science applications. The research of Cheraghali et al. [3] further highlights the application potential of nanoparticles in medicine as MRI contrasting agents by performing an in vitro evaluation of MnZn ferrite nanoparticles coated with polyethylene glycol (PEG). The hierarchical morphology of these nanoparticles, resembling dandelion structures, offers an increased specific surface area, potentially improving the efficacy of contrast enhancement in MRI applications. The study not only compares the morphological and surface characteristics of these nanoparticles to those with a normal structure, but also assesses their cytotoxicity and hemocompatibility.

Improving the synthesis methods to generate biocompatible nanoparticles for use in wound healing applications is also a field of interest. This is demonstrated by the work of Selmani et al. [4], which focuses on exploring improved synthetic methods of Au nanoparticles via the Turkevich method. This research explores the potential of Au nanoparticles to enhance wound healing through their radical scavenging activity, contributing to the development of innovative treatments.

*The use of nanoparticles in optics, electronics, and energy applications* is a subject of heightened interest for fundamental and applied research. The computational study conducted by Domenikou et al. [5] explores the enhancement of nonlinear optical properties near metallic nanoparticles using a polar zinc–phthalocyanine molecule near a gold nanosphere as a model system. The research underscores the impact of nanoparticle proximity on optical rectification coefficients under various external field conditions, providing insights into the design and optimization of photonic materials and devices that exploit the unique electromagnetic interactions between nanoparticles and molecular systems. The experimental work of Shen et al. [6] introduces a novel approach to enhancing the sintering performance of silver nanoparticle pastes through surface modification with organic amines. This method significantly improves the electrical conductivity and mechanical strength of sintered joints, providing a promising solution for high-performance electronic packaging applications. 

High-performance materials are in high demand for energy applications. In this case, nanoparticles can make enormous contributions, as highlighted by the experimental work of Biehler et al. [7]. The authors of this work focused on the use of platinum nanoparticles supported on carbon spheres derived from sugar, a sustainable source, for the efficient generation of hydrogen—a clean energy carrier. 

In addition to bioimaging applications, the design of luminescent materials is also of paramount importance for civil applications; for example, luminescent nanoparticles in pigments for road signs, markings, and lines enhance nighttime visibility and traffic guidance. The study of Cai et al. [8] focuses on improved luminescence persistence by improving the parameters of the synthesis method.

*The application of nanoparticles in environmental applications and sustainability* represents an emerging field with significant advantages over traditional materials. Pauli and Honciuc [9] explored an innovative approach to water purification: their study describes the use of Janus nanoparticles supported by wax colloidosomes for the extraction of metal ions from wastewater. The research highlights the potential of these nanostructured materials to float on water surfaces, efficiently adsorb and recover ions, and withstand multiple extraction cycles, offering a novel and sustainable alternative to traditional ion-exchange technologies. Further, the work of Honciuc et al. [10] proposes the concept of the hydrological mining of Cu(II) ions, with the help of specially designed nanoparticles–hydrogel polymer–microsphere composites capable of floating on water surfaces. The composite material combines nanostructured polymer microspheres with a polyvinyl alcohol (PVA) matrix, demonstrating the ability to adsorb and recover metal ions efficiently. This innovative approach suggests a more environmentally friendly method for extracting valuable metals from water bodies, reducing the need for energy-intensive pumping and processing. 

The realm of nanoparticle applications extends to their potential use in agriculture as effective antifungal agents. For example, the antifungal properties of synthesized copper nanoforms against Colletotrichum gloeosporioides, a plant pathogen, are demonstrated in the experimental study of Vestergaard et al. [11], which reveals the potential of copper nanoparticles to serve as effective antifungal agents in agriculture. This research examines the impact of nanoparticle size, distribution, and oxidation state on their antifungal efficacy, providing valuable insights into the mechanisms of action and suggesting strategies for the sustainable management of plant diseases.

The use of nanoparticles as advanced catalysts represents the traditional realm of nanoparticle applications, which is ever-expanding. The work of Shesterkina et al. [12] demonstrates that bimetallic Pd-Fe/SiO2 catalysts have wide potential practical implications as new non-toxic alternative to the Lindlar catalyst for the selective hydrogenation of triple C≡C bonds in the liquid phase at room temperature. The selective hydrogenation of alkynes is important for the synthesis of pharmaceuticals, vitamins, nutraceuticals, fragrances, etc.

*Creating advanced nanoparticle composites with unique magnetic properties* is a widely pursued application area, as these nanoparticles could be valuable as active components in miniaturized transformers and reactive electronic components, electromagnetic shielding, actuators, spintronics, and beyond. The study of Angotzi et al. [13] examines the formation mechanisms of bi-magnetic core–shell nanoarchitectures, using cobalt ferrite nanoparticles as seeds to grow a manganese ferrite shell, shedding light on the competitive nucleation processes and the impact on magnetic properties. The findings contribute to the development of materials with tailored magnetic behaviors, offering potential applications in data storage and medical imaging. Khairani et al. [14] study the solvent influence on the magnetization and phase of the Fe-Ni alloy nanoparticles. The research provides a detailed analysis of the phases, magnetization, and oxidation levels of nanoparticles synthesized in various solvents, offering insights into the control of nanoparticle characteristics through solvent manipulation.

Further, controlling the morphology, or the shape and structure, of nanoparticles is crucial for optimizing their properties and functionalities. The morphology determines how nanoparticles interact with their environment and, by extension, their effectiveness in a given application. For instance, the shape of a nanoparticle can influence how it is absorbed by cells, its catalytic activity, and its optical properties. This control over morphology allows scientists and engineers to tailor nanoparticles for specific purposes, enhancing their performance and opening up new application possibilities.

Designing nanoparticles with specific morphologies and developing physical synthetic methods to create them are fundamental aspects of nanotechnology research. The ability to design nanoparticles deliberately involves understanding the relationship between the structure of nanoparticles and their properties. This knowledge guides the development of synthetic methods that are not only capable of producing nanoparticles with the desired characteristics, but also do so in a reliable, scalable, and environmentally friendly manner with the help of physical methods. Advancements in synthetic methods are essential for the practical application of nanoparticles, enabling the mass production of nanomaterials with controlled properties and ensuring their widespread use in various industries. Hamdan and Stafford [15] introduce us to a versatile route for the synthesis of metal nanoalloys using a novel spark discharge method for producing metal nanoalloys in a liquid environment, exploiting the interface between two immiscible liquids. The technique enables the synthesis of nanoparticles with controlled composition and embedded in a carbon matrix, opening up new avenues for the creation of nanoalloys with tailored properties for catalysis, plasmonics, and energy conversion applications. The electrochemical method is yet another synthesis method for nanoparticles, as demonstrated by the work of Yuan et al. [16]. This work reports an innovative electrochemical approach for the synthesis of Nb-doped BaTiO_3_ nanoparticles and highlights the ability to control dopant concentrations and achieve high crystallinity under mild conditions. The research emphasizes the significance of alkalinity in the synthesis process, detailing the impact on crystal grain size, distribution, and the potential applications of the resulting nanoparticles in electronic materials, showcasing a novel route for doping and tailoring the properties of nanoceramics. The hydrothermal synthesis of nanoparticles is represented by the work of Milićević et al. [17] that focuses on the hydrothermal synthesis of upconversion nanoparticles doped with ytterbium (Yb^3+^) and thulium (Tm^3+^), aiming to optimize their luminescent properties for applications in bioimaging and security. The study systematically analyzes the effect of dopant concentrations on emission properties, providing insights into the structural and photoluminescent characteristics of these nanoparticles, and suggesting optimal doping levels for enhanced near-infrared emission.

Accompanying the discussion of synthesis and functionalization methods, this Special Issue also highlights the importance of the development of metrology tools for nanoparticles, especially the development of new methods of measuring the nanoparticle surface properties. While monitoring the physicochemical transformation of macroscopic surfaces is trivial, monitoring such changes at the nanoscale is extremely challenging due to the lack of methods and tools. Among these changes in the physicochemical properties of nanoparticles are changes in surface wettability from the liquid of nanoparticles, following a chemical surface modification via physical treatment. Monitoring the change in the wettability of nanoparticles via the contact angle and surface energy is extremely important in predicting the nanoparticles’ behavior in terms of their dispersibility in water and air, pelleting ability, and interaction with solvents or other molecules, and could predict their potential risks toward the environment and living organisms. In this context, a new method for measuring the surface energy of nanoparticles was developed by Honciuc and Negru [18], namely, the NanoTraPPED method. This research offers a novel approach to monitoring the surface energy changes of nanoparticles during functionalization reactions. The study details the application of this method to silica nanoparticles undergoing various surface reactions, providing insights into the physicochemical transformations and the impact of molecular complexity on surface energy. This contribution advances the understanding of nanoparticle functionalization, offering a valuable tool for characterizing surface modifications.

These detailed descriptions offer a comprehensive overview of the contributions to the Special Issue, highlighting the multifaceted nature of nanoparticle research and its potential to address complex challenges across a wide spectrum of scientific and technological domains.
